# Editorial: Pharmacogenetics and pharmacogenomics in Latin America: ethnic variability, new insights in advances and perspectives: a RELIVAF-CYTED initiative, Volume II

**DOI:** 10.3389/fphar.2023.1211712

**Published:** 2023-05-03

**Authors:** Andrés López-Cortés, Patricia Esperón, Matías F. Martínez, María A. Redal, Alberto Lazarowski, Nelson M. Varela, Ismael Lares-Asseff, Luis A. Quiñones

**Affiliations:** ^1^ Cancer Research Group (CRG), Faculty of Medicine, Universidad de Las Américas, Quito, Ecuador; ^2^ Latin American Network for the Implementation and Validation of Clinical Pharmacogenomics Guidelines (RELIVAF-CYTED), Santiago, Chile; ^3^ Molecular Genetics Laboratory, Clinical Biochemistry Department, School of Chemistry, Universidad de la República, Montevideo, Uruguay; ^4^ Laboratory of Chemical Carcinogenesis and Pharmacogenetics, Department of Basic-Clinical Oncology (DOBC), Faculty of Medicine, University of Chile, Santiago, Chile; ^5^ Department of Pharmaceutical Science and Technology, Faculty of Chemical and Pharmaceutical Sciences, University of Chile, Santiago, Chile; ^6^ Genetic Division, Department of Medicine, Faculty of Medicine, Hospital de Clínicas José de San Martín, Buenos Aires University, Buenos Aires, Argentina; ^7^ Clinical Biochemistry Department, Faculty of Pharmacy and Biochemistry, Institute for Research in Physiopathology and Clinical Biochemistry (INFIBIOC), Hospital de Clínicas José de San Martín, University of Buenos Aires, Buenos Aires, Argentina; ^8^ Academy of Genomics and Laboratory of Pharmacogenomics and Molecular Biomedicine, Instituto Politécnico Nacional, CIIDIR-Unidad Durango, Durango, Mexico

**Keywords:** pharmacogenetics, Latin America and the Caribbean, clinical guidelines, pharmacogenomics, drugs

Pharmacogenetics and pharmacogenomics (PGx) have revolutionized our understanding of the relationship between genes and drug response, leading to improved therapeutic regimens in terms of efficacy and safety (Pirmohamed, 2023; Sadee et al., 2023). Although international efforts have developed clinical guidelines for daily practice, most research originates from the United States and Europe, often excluding or generalizing the Latin American population (Barbarino et al., 2018; Relling et al., 2020). Recently, scientific societies have aimed to close this gap, but Latin America faces unique challenges, such as its vast genetic diversity, with distinct frequencies or polymorphisms compared to other regions (Karczewski et al., 2020). Moreover, there is a lack of high-quality, population-focused research on gene-drug response relationships and knowledge of frequency data in the region. These factors collectively impede the implementation of PGx in clinical practice throughout Latin America.

In this context, this Research Topic includes four scientific articles related to: a reliable and clinically useful method for estimating tacrolimus exposure in Chilean pediatric kidney transplant recipients using a limited-sampling strategy; the discovery of pharmacogenomic profiles for anesthesia drugs in Colombians, emphasizing the importance of next-generation sequencing data in personalized medicine; a genome-wide data integration approach to prioritize drug targets for protozoan parasites responsible for global diseases, validating known candidates and uncovering new potential target; and an overview of pharmacogenomic progress in Latin America and the Caribbean (LAC), identifying barriers to clinical implementation and highlighting the region’s growing awareness and potential for future clinical applications.

Firstly, Galvez et al. investigated the effectiveness of limited-sampling strategies (LSS) for estimating the area under the curve (AUC) and CYP3A5 genotype in Chilean pediatric kidney transplant recipients who were using extended-release tacrolimus (TAC), an immunosuppressive drug mainly metabolized by CYP3A5 and monitored by trough levels (C_0_). However, this method is not very reliable. The study compared daily TAC dose and AUC_(0-24)_ normalized by dose between CYP3A5 expressors and non-expressors and found significant differences (1701.9 vs. 2718.1 ng*h/mL/mg/kg, *p* < 0.05). The model that included C_0_, C_1_, and C_4_ showed the best performance in predicting LSS-AUC_(0-24)_ with an r^2^ of 0.8765 and the lowest precision error (7.1% ± 6.4%). The study concludes that using three time-points for estimating LSS-AUC_(0-24)_ is a clinically useful and advisable option for pediatric kidney transplant recipients using extended-release TAC.

Secondly, Parada-Márquez et al. provided insight into the impact of genetic variability on adverse drug reactions (ADRs) in response to common anesthesia drugs in the Colombian population. The study included 625 healthy Colombian individuals and focused on 14 genes related to the metabolism of analgesic and anesthetic drugs. Whole-exome sequencing (WES) was used to identify rare and common variants, which were then assessed for their functional impact using an optimized prediction framework (OPF). The researchers identified 148 molecular variants potentially related to variability in the therapeutic response, with 83.1% being rare and novel missense variants classified as pathogenic, 5.4% loss-of-function (LoF), 2.7% causing potential splicing alterations, and 8.8% being actionable or informative pharmacogenetic variants. The study revealed a unique pharmacogenomic profile for anesthesia drugs in the Colombian population, emphasizing the importance of incorporating next-generation sequencing data in pharmacogenomic approaches and personalized medicine.

Thirdly, Rivara-Espasandín et al. concentrated on devising innovative control strategies for *Trypanosoma cruzi*, *Trypanosoma brucei*, and *Leishmania* species (TriTryps), a collection of protozoan parasites responsible for diseases that impact millions of people globally. Current treatments offer limited effectiveness and come with severe side effects. The researchers employed a comprehensive genome-wide data integration method that encompassed genomic, transcriptomic, metabolic, and protein structural information to prioritize drug targets. The subsequent ranked list features shared proteins with diverse biological functions, essential for the parasites’ survival or growth, oxidative stress-related enzymes, virulence factors, and proteins unique to these parasites. This methodology validates previously reported candidates and unveils new potential targets for drug discovery.

Finally, Salas-Hernández et al. reviewed and analyzed pharmacogenomic knowledge within the Latin American and the Caribbean scientific and clinical communities, examining potential barriers to the clinical implementation. Additionally, they analyzed a paired list of 54 genes/drugs associations to determine relationships between biomarkers and responses to genomic medicine. The regional structured survey was compared to a previous survey conducted in 2014 to assess progress in the region (Quinones et al., 2014). The results indicated that LAC countries have contributed 3.44% of total publications and 2.45% of the PGx-related clinical trials worldwide thus far. Despite continuous effort in the region over the last decade, six major groups of barriers were identified, with the most relevant being the “need for guidelines, processes, and protocols for the clinical application of pharmacogenetics/pharmacogenomics.” Based on the survey results, the highest-ranked (96%-99%) gene/drug pairs perceived as important were CYP2D6/tamoxifen, CYP3A5/tacrolimus, CYP2D6/opioids, DPYD/fluoropyrimidines, TMPT/thiopurines, CYP2D6/tricyclic antidepressants, CYP2C19/tricyclic antidepressants, NUDT15/thiopurines, CYP2B6/efavirenz, and CYP2C19/clopidogrel. In conclusion, although the global contribution of LAC countries remains low in the PGx field, a significant improvement has been observed in the region. The perception of the usefulness of PGx tests in the biomedical community has drastically changed, raising awareness among physicians and suggesting a promising future for clinical applications of PGx in LAC.
